# Integrated Multi-Omics Analysis Identified PTPRG and CHL1 as Key Regulators of Immunophenotypes in Clear Cell Renal Cell Carcinoma(ccRCC)

**DOI:** 10.3389/fonc.2022.832027

**Published:** 2022-03-30

**Authors:** Xing Zeng, Le Li, Zhiquan Hu, Dan Peng

**Affiliations:** ^1^ Department of Urology, Tongji Hospital, Tongji Medical College, Huazhong University of Science and Technology, Wuhan, China; ^2^ Department of Nuclear Medicine, Tongji Hospital, Tongji Medical College, Huazhong University of Science and Technology, Wuhan, China

**Keywords:** multi-omics study, bioinformatics, ccRCC kidney cancer, prognosis model, immunophenotype

## Abstract

Despite the increasing importance and status of immune checkpoint blockade (ICB), little is known about the underlying molecular mechanisms determining the target clear cell renal cell carcinoma (ccRCC) population. In this study, we screened out 6 immune cells strongly correlated with expression levels of PD-L1 and IFN-γ based on the ccRCC samples extracted from GSE and TCGA data sets. By performing unsupervised clustering and lasso regression analysis, we grouped the ccRCC into 4 clusters and selected the two most distinct sub-clusters for further investigation—cluster A1 and B1. Next, we compared the two clusters in terms of mRNA, somatic mutations, copy number variations, DNA methylation, miRNA, lncRNA and constructed the differentially expressed genes (DEGs) hub by combing together the previous results at levels of DNA methylation, miRNA, and lncRNA. PTPRG and CHL1 were identified as key nodes in the regulation hub of immunophenotypes in ccRCC patients. Finally, we established the prognosis model by using Lasso-Cox regression and Kaplan–Meier analysis, recognizing WNT2, C17orf66, and PAEP as independent significant risk factors while IRF4 as an independent protective factor.

## Introduction

Kidney cancer remains the third most frequent urinary carcinomas worldwide, with an estimated number of 431,288 new cases and 179,368 deaths globally in 2020 (1). Clear cell renal cell carcinoma (ccRCC), accounting for more than 70% of all RCC cases, is characterized by early deficiency of the von Hippel–Lindau tumor-suppressor gene (VHL) in a majority (60–80%) of neoplasms (2). It has been demonstrated that immune checkpoint inhibitors (ICI) such as nivolumab prolong the overall survival of a subgroup of metastatic ccRCC patients through inhibition of PD-L1-mediated signaling (3). Several studies pointed out distinct subgroups of genomic landscapes and immune phenotypes might be responsible for heterogeneous responses to PD-1 therapy among individual cancer patients (4–6). Miao et al. (7) performed whole-exome sequencing of metastatic ccRCC and proposed that PBRM1 loss in ccRCC may play an important role in the formation of differentiated immune expression landscape and subgroup-specific tumor immune infiltrating microenvironment influencing responsiveness to immune checkpoint therapy.

IFN-γ is a cytokine that plays a critical role in tumor growth and the foundation of tumor microenvironment. An increasing number of studies reveal the correlation of high IFN-γ levels with accelerated lymphocyte infiltration and the clinical benefit of PD1/PD-L1 immune checkpoint inhibition therapy (8, 9). Nevertheless, more accurate and dependable models predicting responsiveness to ICI therapy are needed due to the heterogeneities and complex nature of cancer.

Recently, T cell infiltration score was found to be closely correlated with prognosis and response to immune therapy in ccRCC patients (10–12), and three immune molecular phenotypes with distinct prognostic features were identified (13). The underlying mechanism driving the establishment of the immunophenotype subgroups and further combination of prognostic value with tumor immune infiltration through systemic bioinformatics, however, has not yet been fully clarified.

In this study, we sought to report a detailed integrative analysis of multi-omics to determine the subgroups of immunophenotypes of ccRCC based on the expression levels of PD-1/IFN-γ and relative contents of infiltrating immune cells, and subsequently identify the key molecules of the DEGs-hub responsible for driving the formation of high/low cytotoxic tumor micro-environment in ccRCC patients with better/poorer overall survival. Major deliverables are expected to take a step forward in elaborating the underlying mechanisms of variant responsiveness to ICI and preliminarily stratify patients before treatments to identify optimal schedules.

## Materials and Methods

### Data Acquisition and Processing

Transcriptome data of ccRCC were downloaded from the Gene Expression Omnibus (GSE66270 (n = 28), GSE53757 (n = 144), GSE36895 (n = 76), GSE76351 (n = 24)) and the website UCSC xena GDC TCGA KIRC (https://xenabrowser.net/datapages/) in August 2020. The gene expression data from the TCGA were of the HTSeq-Counts type of 607 cases, namely, 535 tumor samples and 72 normal samples. After removing replicate samples from the same patients, 527 tumor samples and 72 normal samples were obtained. Mutation data were downloaded from the UCSC xena TCGA hub KIRC of the MC3 public version (n = 368) and processed with the VarScan software, and then the Mutation Annotation Format (MAF) of somatic variants were analyzed with R package “maftools” (14) (https://xenabrowser.net/datapages/). CNV data in GISTIC-focal score by gene (n = 536) were downloaded from the UCSC xena GDC TCGA KIRC (https://xenabrowser.net/datapages/). Illumina Human Methylation450k (n = 480) data were downloaded from the UCSC xena TCGA hub KIRC. Considering the miRNA data downloaded directly from the UCSC xena were in the type of log2(RPM + 1), we used the R package TCGAbiolinks (15) to download these data (n = 592) in the Count form as recommended by GDC. Protein data (n = 454) were downloaded from the UCSC xena RPPA TCGA hub (https://xenabrowser.net/datapages/). Survival data (n = 979) were downloaded from the UCSC xena GDC TCGA KIRC (https://xenabrowser.net/datapages/).

### Cibersort Immune Cell Scores and Spearman Correlation Analysis

The Cibersort (cell-type identification by estimating relative subsets of RNA transcripts, https://cibersort.stanford.edu/) deconvolution algorithm was used to calculate the relative contents of 22 kinds of immune cells (LM22 gene signature) and determine immune scores (model = relative, permutation = 1,000) for ccRCC samples downloaded from the GEO and TCGA databases. By applying the ESTIMATE algorithm to the matrix data normalized with the limma R package (version 3.5.2), we extracted PD-L1 and IFN-γ gene expression (gene CD247, IFN-γ) and subsequently calculated the Spearman correlation between the contents of immune cells and the expression levels of PD-L1 and IFN-γ genes.

### Cluster Analysis

The renal clear cell carcinoma samples were filtered out according to the annotation information of GEO samples and the sample name ID of TCGA samples. Lasso was applied to prioritize the most relevant tumor-infiltrating immune cells related to PD-L1 and IFN-γ expression using the R package “glmnet”. Then, based on the contents of these selected immune cells, we used Euclidean distance and Ward (unsquared distances) linkage for unsupervised clustering and created heat maps with hierarchical trees. The log-rank test was carried out to compare Kaplan–Meier curves between immune clusters.

### Somatic Mutation Analysis

Candidate genes of the TCGA-KIRC with a MutSig (version 2.0) q-value <0.05 and a somatic mutation frequency >5% were taken into consideration to compare their distribution. Mutational burden (Total number of mutations in exon regions/30 M) and mutational landscape among all clusters were calculated to record the frequency of mutation, followed by the Fisher test aimed at calculating mutational exclusion and co-existence events among different subtypes (significant = unadjusted *p-*value <0.05).

### Gene Expression Analysis

Using R package EnsDb.Hsapiens.v75 and DESeq2, the normalized matrix data of gene expression variations (mRNA data downloaded as HTSeq-Counts type) among distinct clusters were analyzed with the filtering cut-off set as unadjusted *p <*0.05, |log2(foldchange)| >log2(1.5) using Wald test. Mutual connections among DEGs were indicated with Cytoscape (https://cytoscape.org/). Kyoto Encyclopedia of Genes (KEGG) pathway gene set enrichment analyses were subsequently carried out using the R package “Clusterprofiler”.

### Copy Number Variation Analysis

With IGV_2.4.19 (Integrative Genomics Viewer_2.4.19), copy number variations among distinct immune clusters were illustrated in the CNV summary plots.

### LncRNA and miRNA Variation Analysis

Differentiated expression analysis was carried out using R package DESeq2 with the cut-off set as adjusted *p <*0.05, |log2(foldchange)| >log2(1.5), and subsequent volcano plots were generated. For the upregulated genes in the immune subgroups as defined above, the responding mRNA–miRNA–lncRNA hub was created using the following websites: mircode (http://www.mircode.org/), miRTarBase (http://mirdb.org/), and TargetScan (http://www.targetscan.org/).

### Methylation Variation Analysis

Methylation variation analysis was carried out using R package champ (16) with adjusted *p*-value < 0.05, |log2(fold change) | > 0.15 as the filtering cut-off.

### Lasso-Cox Regression and Prognostic Model

Only KIRC samples with complete clinical prognostic data were included in the survival analysis, and univariate logistic regression analyses were first performed for the differentiated expressed genes between distinct immune clusters using coxph function. Then, lasso regression was applied to screen out the most significant candidate genes to build a multivariate logistic regression prognostic model and coefficients for each covariate were determined by the multivariable Cox regression model. The cut-off of risk score was set as the turning point of the ROC curve and thereby patients were divided into the high-risk group (greater than cut-off value) and the low-risk group (smaller than cut-off value). Kaplan–Meier analysis was carried out with R package “survminer”, a heatmap was generated with “pheatmap” and other corresponding figures were generated with “ggplot2”.

### Renal Cancer Cell Lines and Transfection

Two human renal cancer cell lines 786O and Caki-1 were gifts from Doctor Zhao and were cultured in DMEM containing 10% FBS and penicillin–streptomycin. Transfection of CHL1-pMYs-IP and sh1/sh2/scramble-pMXs-IP was performed using Lipo3000 (Invitrogen, Carlsbad, CA, USA) following recommended protocol.

### Cell Proliferation Assay

Cell proliferation was examined by Bromodeoxyuridine (BrdU, Sigma-Aldrich) assay. Approximately 3.5 ∗ 10^4^ of CHL1-transduced, sh1 and the control empty vector-transduced cells were respectively plated on 24 well plate triplicate. The morphology and total numbers of the cells were analyzed on days 0, 1, 2, and 3 after adding 10 uM BrdU to these cells and incubating for another 4 h. Then, these cells were washed three times by PBS and 300 μl anti-BrdU (ab1893, Abcam, Cambridge, UK, dilution of 1:1,000) was supplemented to these cells overnight at room temperature. Total cell counts were obtained by using a hemocytometer under a microscope (Zeiss, Heidenheim, Germany).

### Cell Apoptosis Assay

After transfection, early and late cell apoptosis were analyzed by using flow cytometry assay (Becton Dickinson) after 48 h. Briefly, cells were washed twice in PBS and re-suspended in 100 μl binding buffer. Then, these cells were stained with 5 μl Annexin V-FITC and 10 μl PI (Santa Cruz Biotechnology, CA) for 10 min in a dark place at room temperature. Flow cytometry analysis was performed by a FACS can (Beckman Coulter, Fullerton, CA, USA). The data were analyzed by using FlowJo software (Treestar, Ashland, OR, USA). More information were clarified in Supplementary files.

### Statistics

All the data were statistically analyzed using the *Stata* version 12.1(*Stata* Corp.) and R software (version 3.5.2). *p*-value <0.05 was considered statistically significant.

## Results

**Figure 1:**
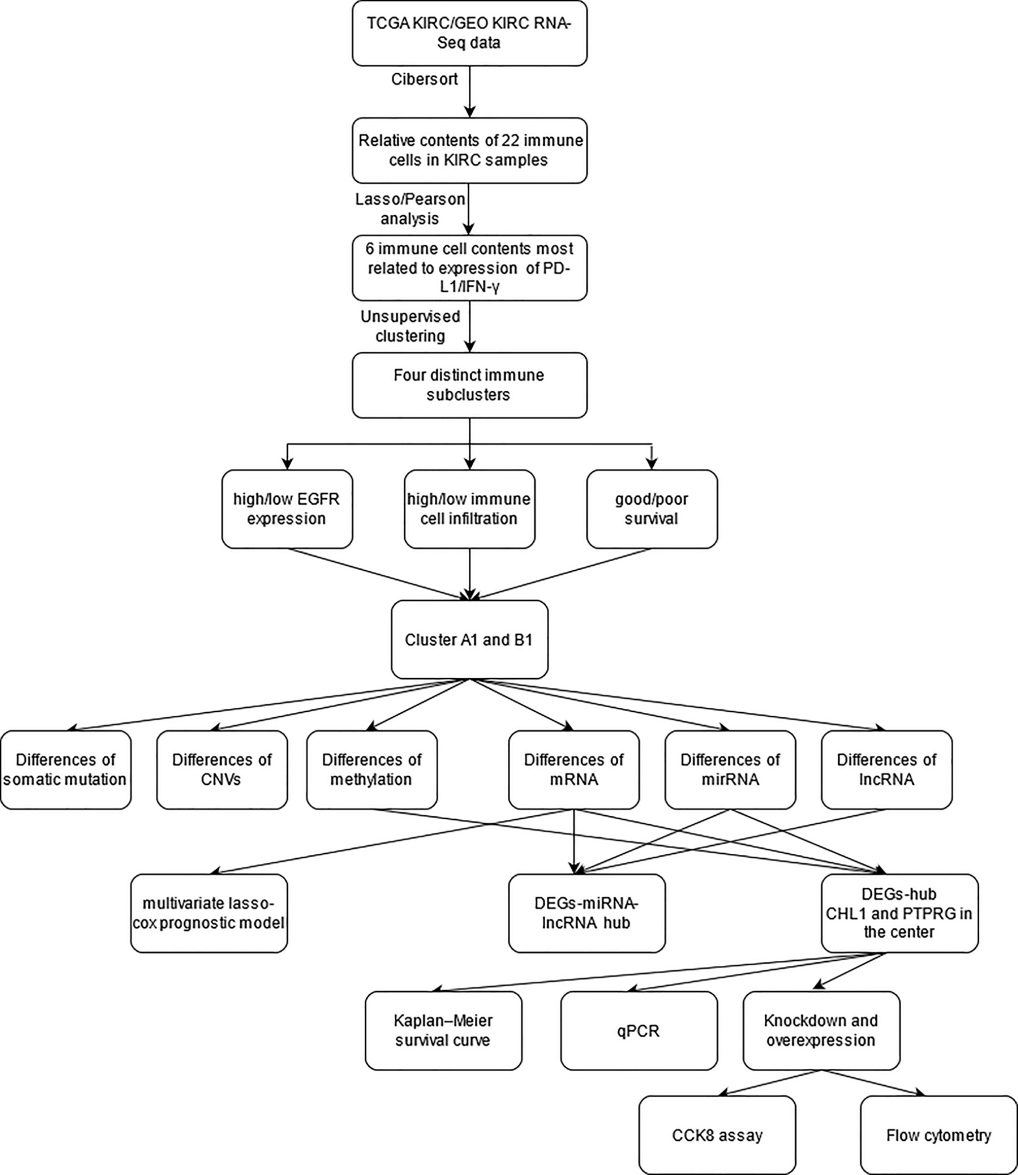
The flow chart of the study strategy.

The study strategy was indicated in the flowchart (**Figure 1**).

### Correlation of 22 Kinds of Immune Cell Contents and Expression of PD-L1, IFN-γ Genes

**Figure 2:**
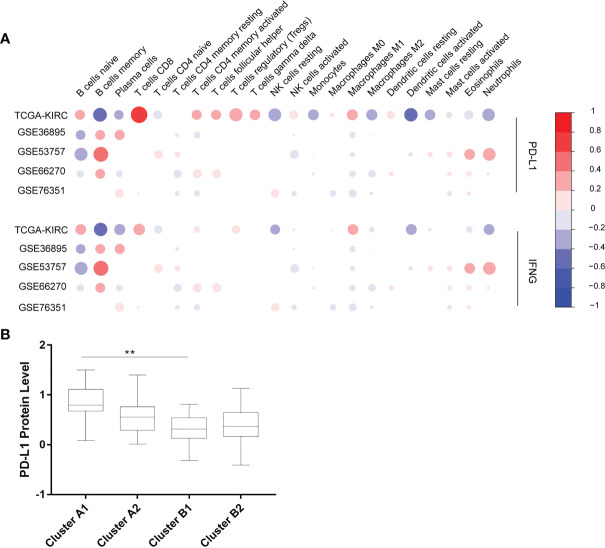
**(A)** Correlation between 22 immune cells contents and expression levels of PD-L1 and IFN-γ. The abscissa indicates 22 kinds immune cells while the ordinate represents 5 independent datasets of KIRC. Red means positively related; blue means negatively related; size of circles and shades of colors mean magnitude of correlation. **(B)** PD-L1 expression levels of 4 immune sub-clusters. **p < 0.001.

The Cibersort was used to calculate the relative contents of 22 kinds of immune cells in five independent cohorts. The data with *p*-values greater than 0.05 was removed. Six immune cells (CD8^+^ T cells, CD4^+^ memory resting T cells, CD4^+^ memory activated T cells, follicular helper T cells, activated NK cells, M2 macrophages) were selected for succeeding analysis, which were significantly correlated with the expression of PD-L1 and IFN-γ in the 5 cohorts above (**Figure 2A**).

### Immunophenotypic Characterization

**Figure 3:**
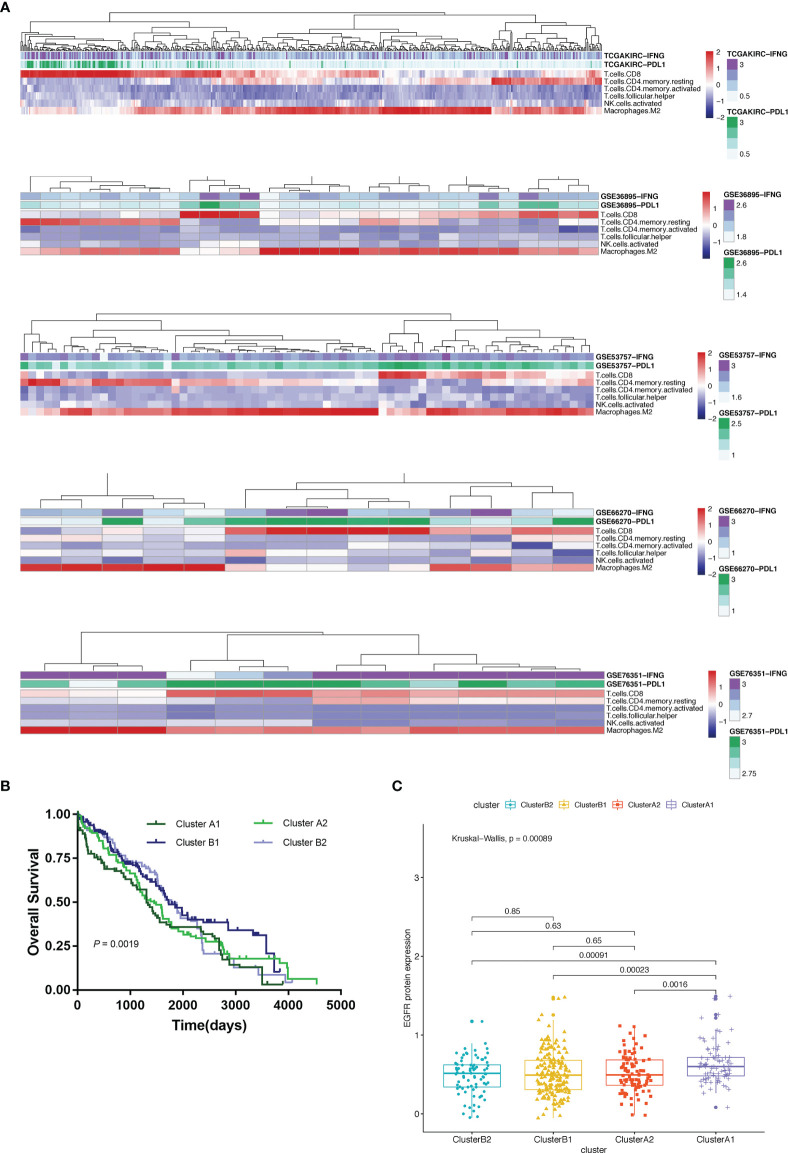
**(A)** Heat map depicting unsupervised clustering of cohorts from the GEO and TCGA-KIRC based on the contents of 6 kinds immune cells. **(B)** Overall survival of 4 immune clusters. **(C)** A boxplot showing expression levels of EGFR among 4 immune clusters.

Using unsupervised hierarchical clustering based on the expression levels of PD-L1 and IFN-γ and also the six selected immune cell subtypes, we divided these samples into two main clusters: cluster A (higher expression of PD-L1 and IFN-γ) and cluster B (lower PD-L1 transcripts) (**Figure 2B**). Moreover, the M2 macrophages and CD4^+^ memory resting T cells were significantly enriched in cluster B instead of in cluster A while CD8^+^ T cells exhibited higher infiltration rates in cluster A than in cluster B (**Figure 3A**). According to these characteristic variations, we further divided cluster A into subcluster A1 and A2 while cluster B into B1 and B2 and the differences of PD-L1, IFN-γ expression levels and immune cells infiltration rates were even greater between subcluster A1 and B1 (**Figures 2B** and **3A**). Considering previous studies demonstrated M2 macrophages were negatively related to overall survival and CD8^+^ T cells played a major role in anti-tumor immunity (17–19), we thus defined subcluster A1 as high cytotoxic immune phenotype while subcluster B1 as low cytotoxic immune phenotype. In our data, the contents of CD8^+^ T cells are negatively correlated with those of Macrophages M2, while in several previous studies the contents of CD8^+^ T cells were believed to predict a better survival benefit in multiple tumor types, Macrophages, however, in the opposite side (20–23). In line with this, patients in cluster A1 exhibited significantly better overall survival than patients in cluster B1 (**Figure 3B**). Besides, we also investigated the protein levels of epidermal growth factor receptor (EGFR) among the four clusters since an increasing body of evidence highlighted its unique role in driving the stratification of landscapes of immune infiltration in various types of cancers (24–26) and found significantly higher expression of EGFR in cluster A1 than in cluster B1 or B2 (**Figure 3C**). Accordingly, we selected clusters A1 and B1 for further analysis and attempted to underline the mechanisms driving distinct immune subtypes in ccRCC.

### Somatic Mutation Landscape of Different Immune Phenotypes

**Figure 4:**
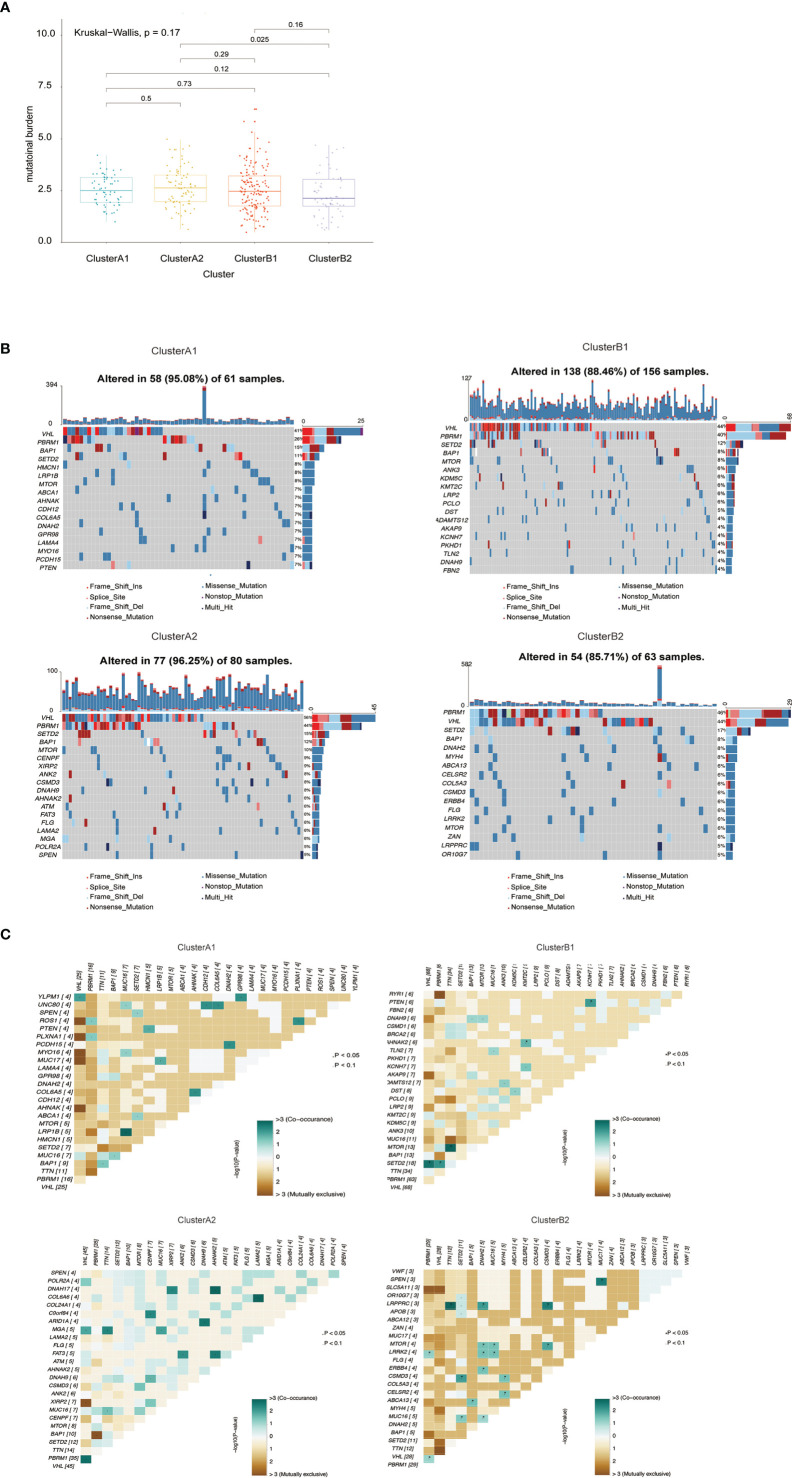
**(A)** Barplot of mutation load among various immune clusters. **(B)** Waterfall plot of mutational profiles among various immune clusters. **(C)** Overview of mutational coexistence and mutual exclusion time.

The quality and quantity of somatic mutations of all immune phenotypes were assessed based on the TCGA-KIRC cohorts to reveal an association between mutations and immune alteration. In terms of total mutational loads, clusters A1 and B1 reported no difference while cluster A2 indicated significantly higher mutation loads than B2 (**Figure 4A**). Although several unique genes with high mutation frequency were observed respectively in cluster A1 (HMCN1, LRP1B), B1 (ANK3, KDM5C), A2 (CENPF, XIRP2) and B2 (DNAH2, MYH4), VHL, PBRM1, SETD2 and BAP1 ranked the top four most frequently mutated genes in all clusters, unraveling their irreplaceable position in driver mutations (**Figure 4B**). We also calculated mutational co-existing and exclusive events among the four sub-clusters (**Figure 4C**), since these events were possibly strong predictors of oncogenesis and progression of ccRCC (27, 28). Of note, the exclusiveness of PBRM1 and BAP1 could be observed in sub-cluster A1 while the co-existence of VHL and SETD2 occurred in sub-cluster B1 (*p <*0.05).

### Differences in mRNA Expression Related to Immunophenotypes

**Figure 5:**
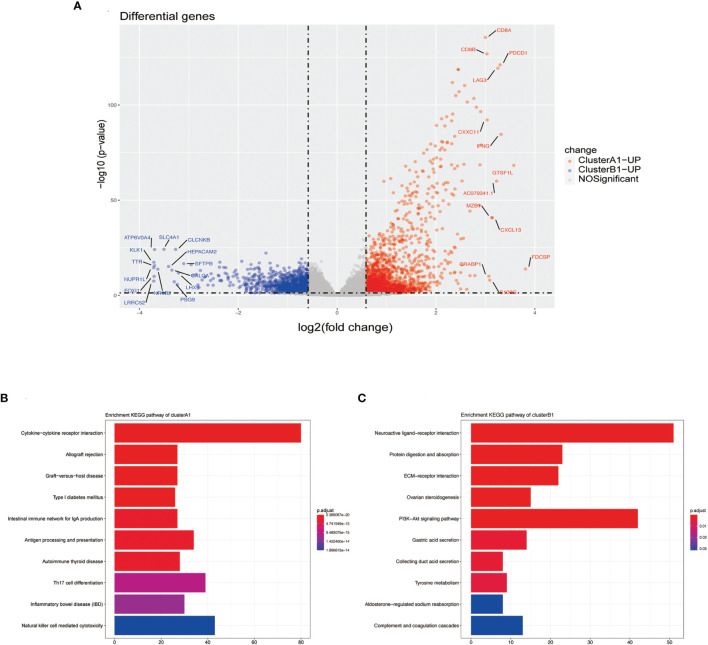
**(A)** Volcano plots DGEs. **(B)** Barplot of upregulated genes in cluster A1 by using the KEGG pathway enrichment analysis of DEGs. **(C)** Barplot of upregulated genes in cluster B1 by the using KEGG pathway enrichment analysis of DEGs.

Differentiated analysis for the expression of mRNA between sub-clusters A1 and B1 was conducted after applying the filtering criteria (fold change ≥ 1.5, *p-*value <0.05) and identified 1,890 upregulated genes in A1 and 1155 upregulated genes in B1 (**Figure 5A**). After adjustment of the filtering criteria to fold change ≥8, *p-*value <0.0001, significantly up-expressed genes in A1 included CD8A, CD8B, LAG3, IFN-γ while SLC4A1 is up-expressed in B1. Notably, pathways related to cytokine–cytokine receptor interaction, allograft rejection, graft-versus-host disease were activated in A1 while pathways related to neuroactive ligand–receptor interaction and protein digestion and absorption were activated in B1, in accordance with our previous results defining A1 as high cytotoxic immune phenotype and B1 as low cytotoxic immune phenotype (**Figures 5B, C**).

### Differences in Genetic Copy Number in Immune Subtypes

**Figure 6:**
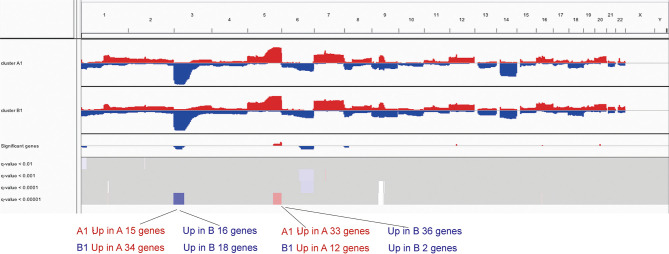
IGV profiles for copy number variations between clusters A1 and B1.

To further investigate the differences in copy number, we conducted *GISTIC*-2.0 analysis for A1 and B1 and found 15 significantly amplified regions and 6 significantly deleted regions in A1 while 9 and 4 in B1 correspondingly (**Figure 6**). Significantly altered regions were divided into 4 levels as follows: stage 1, *p <*0.01; stage 2, *p <*0.001; stage 3, *p <*0.0001; and stage 4, *p <*0.00001. Taking the intersection of genes included in these regions and differentially expressed genes previously obtained, we could see that significantly deleted genes of A1 and B1 exist in neighboring instead of identical regions on chromosome 3 (**Supplementary Table 1**). Besides, copy number distinctions between A1 and B1 were also identified in chromosome 5 and we hereby recognized several vital genes among them: CD74, NKX2-5, EGR1, and FOXI1 (**Supplementary Table 1**).

### Differences in DNA Methylation, miRNA and lncRNA in Immune Subtypes

**Figure 7:**
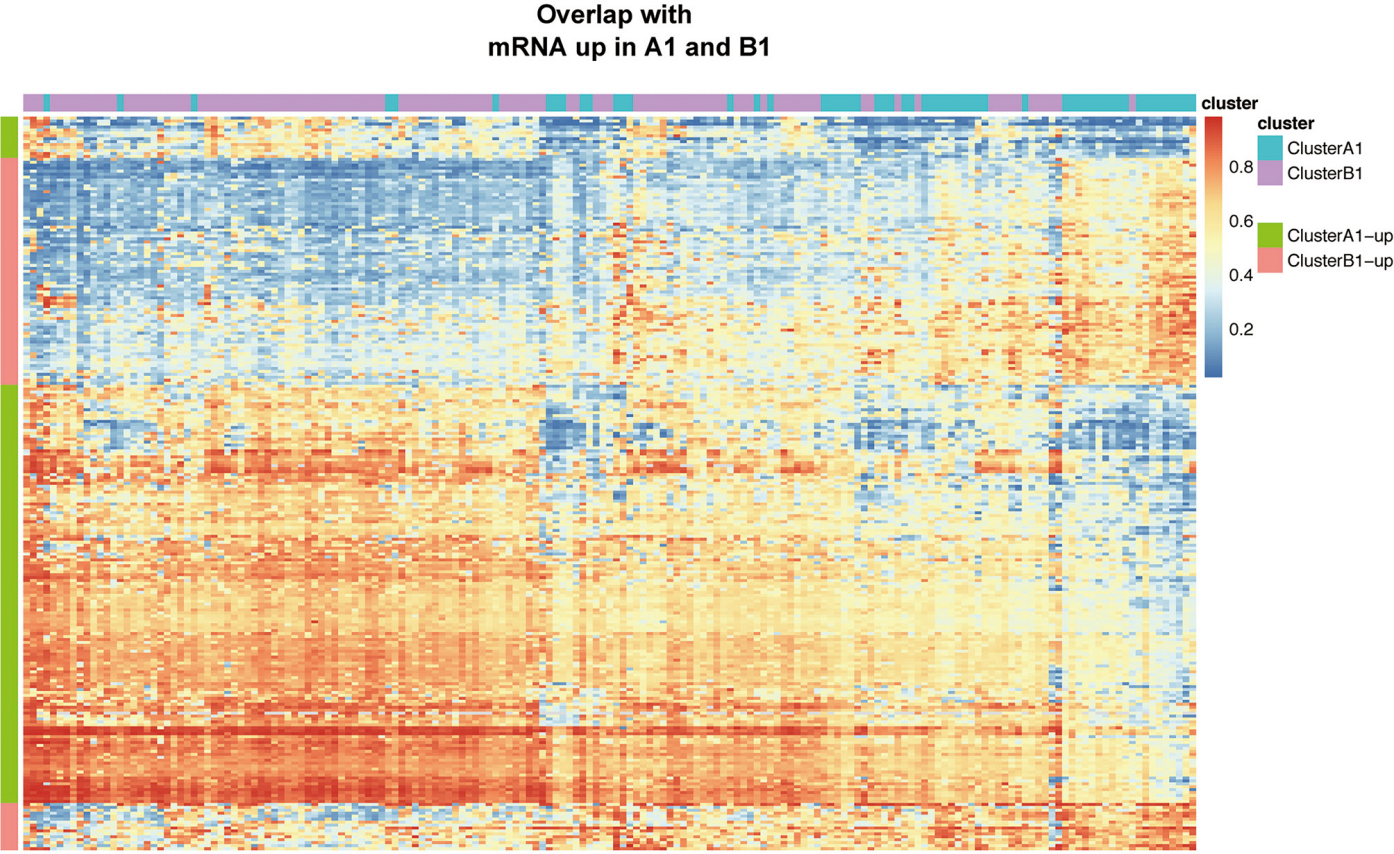
Heat map showing intersection of differential DNA methylation and DEGs between clusters A1 and B1.

Based on the filtering standard of (|log FC|>0.15, adjust *p <*0.05), 523 differentially upregulated hypermethylated probes in A1 and 1,456 differentially upregulated hypermethylated probes in B1 were identified. Among them, 156 probes with higher beta values were found to be associated with 94 differentially upregulated genes in cluster A1 while 93 probes with higher beta values were related with 72 differentially upregulated genes in cluster B1 (**Figure 7**).

**Figure 8:**
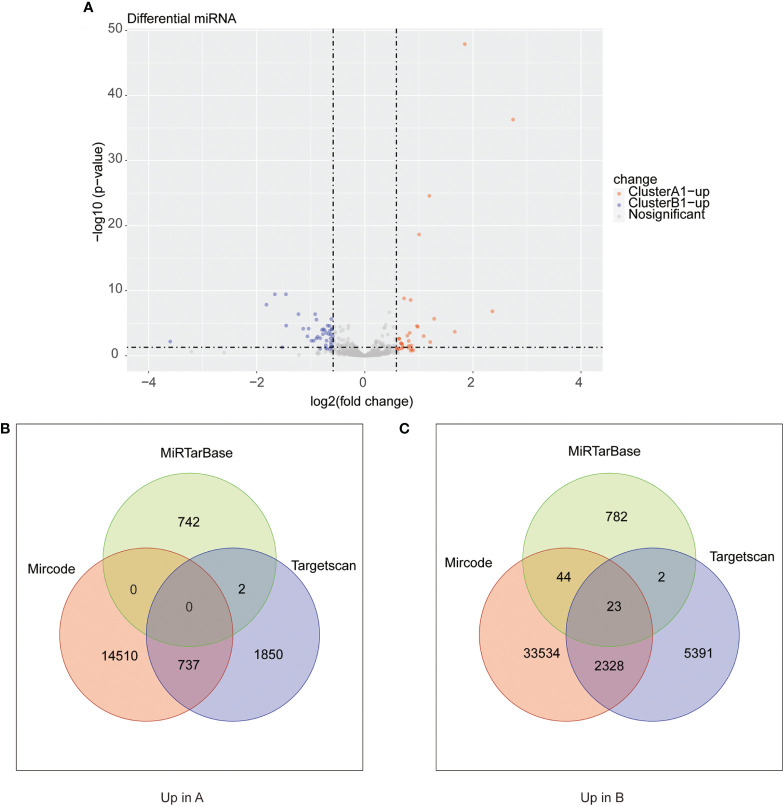
**(A)** Heat maps of differential miRNA between clusters A1 and B1. **(B, C)** Venn diagram showing upregulated mRNA in cluster A (diagram B) and cluster B (diagram C) with miRNA–mRNA interaction pairs in datasets of mircode, miRTarBase, and TargetScan.

We next searched the miRNA–mRNA relationship group based on the three databases—mircode miRTarBase and Targetscan under the premise that the corresponding mRNA at least appears in 2 out of the three databases. For the 33 upregulated genes in A1, 739 miRNA–mRNA relationship groups were discovered while for the 47 upregulated genes in B1, 2,443 miRNA–mRNA relationship groups were discovered (**Figure 8**).

**Figure 9:**
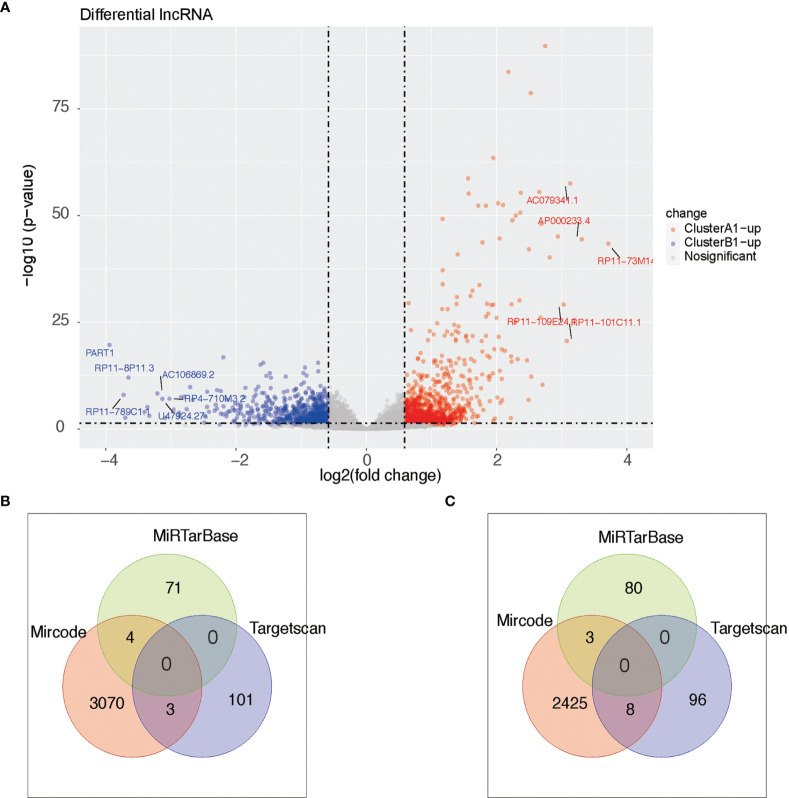
**(A)** Heat maps of differential lncRNA between clusters A1 and B1. **(B, C)** Venn diagram showing upregulated lncRNA in cluster A (diagram B) and cluster B (diagram C) with miRNA–lncRNA interaction pairs in datasets of mircode, miRTarBase, and TargetScan.

As reported above, we found 1,288 upregulated lncRNA in A1 and 754 upregulated lncRNA in B1. Likewise, we searched the miRNA–lncRNA relationship groups based on the mircode, miRTarBase, TargetScan database, then identifying 7 miRNA–lncRNA relationship groups for the upregulated lncRNA in A1 and 11 miRNA–lncRNA relationship groups for the upregulated lncRNA in B1 (**Figure 9**).

### Construction of the mRNA–miRNA–lncRNA Regulation Hub

**Figure 10:**
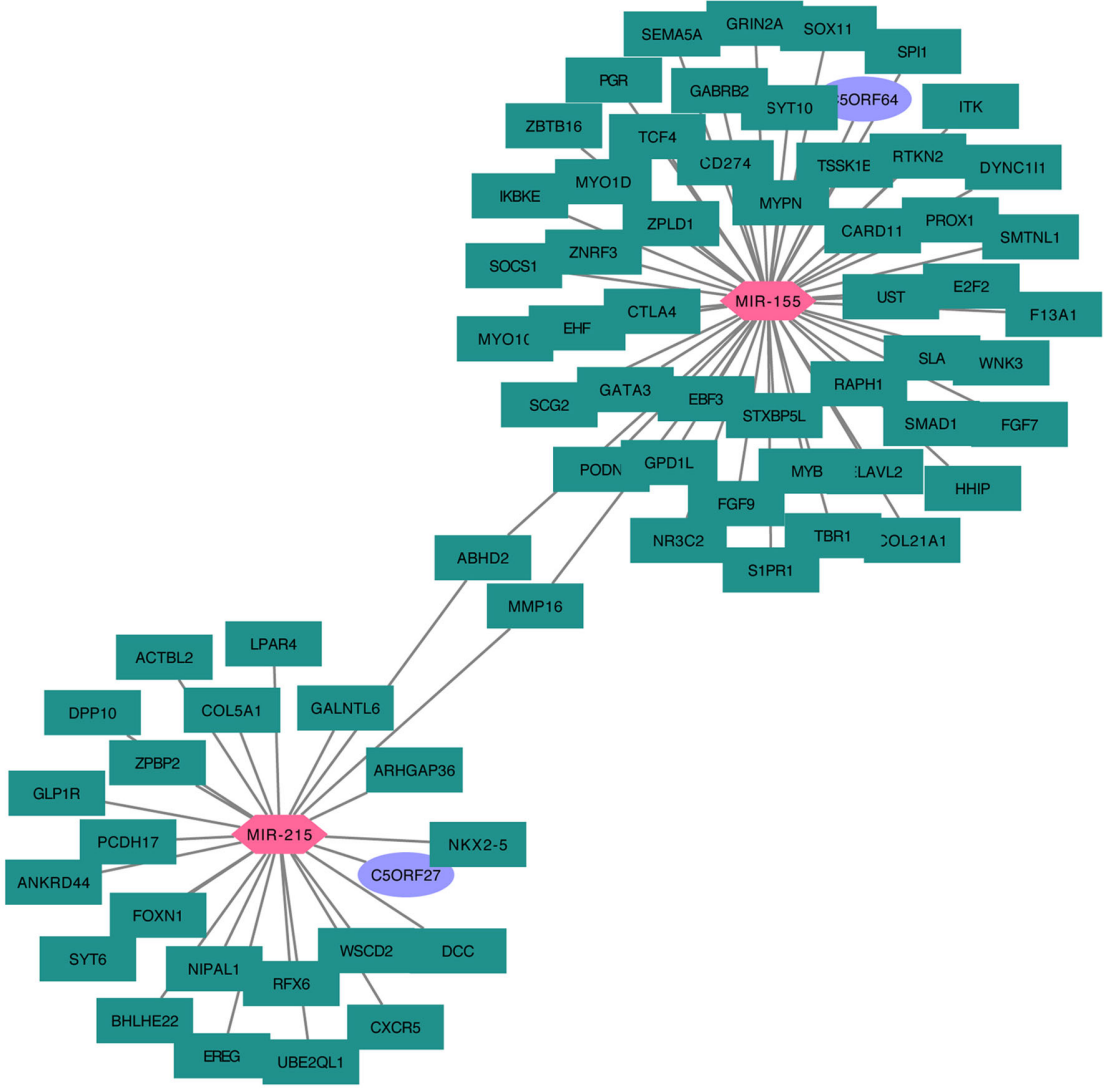
Cytoskype network diagram of DEGs–miRNA–lncRNA.

Taking the mRNA–miRNA–lncRNA relationship groups together, two significant miRNA, miR-155 and miR-215, were identified as key nodes in the center of the regulation hub associated with multiple differentially distributed mRNA and lncRNA in A1 and B1 (**Figure 10**).

### Construction of Differentially Expressed Genes (DEGs) Hub

**Figure 11:**
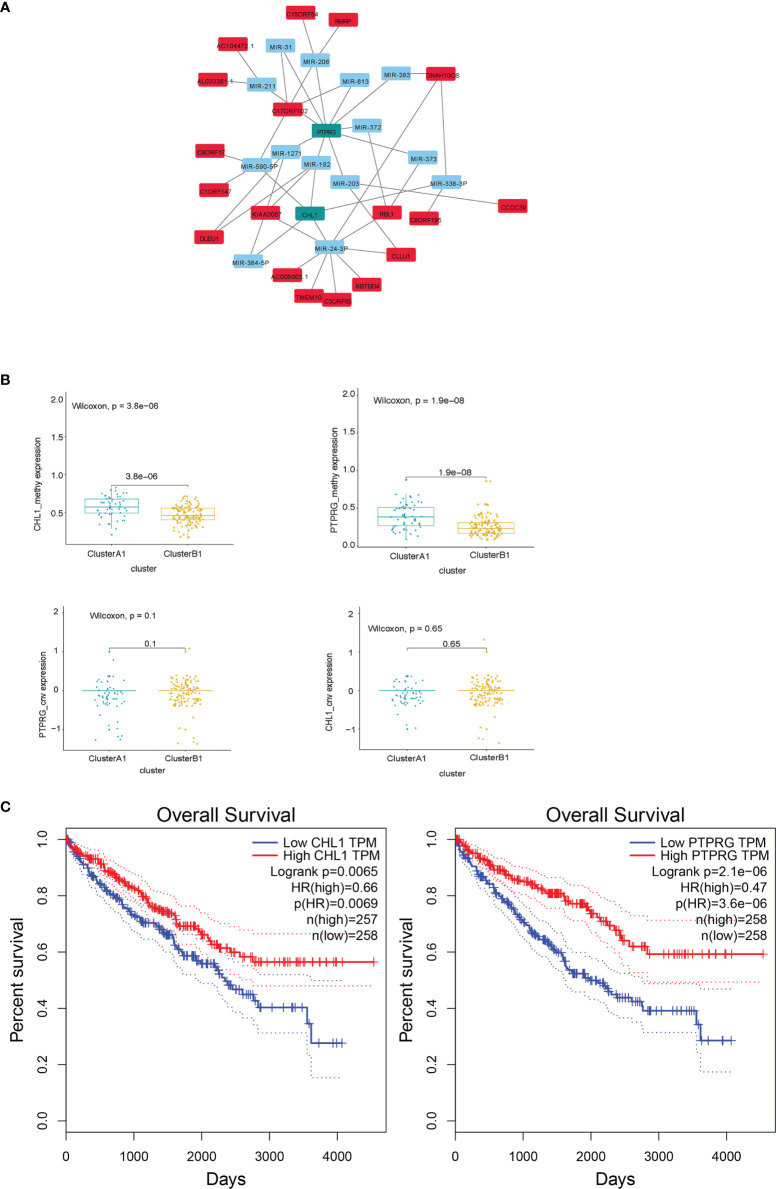
**(A)** DEG hub genes diagram. **(B)** Methylation and copy number variations differences of PTPRG and CHL1 between clusters A1 and B1. **(C)** Survival curves using univariate Kaplan–Meier estimates based on expression levels of CHL1 and PTPRG in TCGA-KIRC cohorts with the online tool Gepia.

Since similar patterns of copy number variations between cluster A1 and B1 were discovered and limited numbers of genes were obtained by comparing distinct lncRNA–mRNA groups previously, we did not include the results of these two parts in the DEGs hub construction. Taking the intersection of differentially expressed genes from the results obtained above, namely, the genes with a differentiated expression of mRNA, the genes with differentiated methylation and the genes with differentiated miRNA–mRNA relationships groups, we screened out 40 genes altogether, among which 18 were upregulated in A1 while 22 were upregulated in B1. We next conducted PPI analysis for these 40 genes in the STRING database and drew a network (**Figure 11A**). It was shown that the methylation expressions in A1 were both higher than those in B1 (**Figure 11B**), the RNA expressions in A1 were both lower than those in B1 (data not shown) while the CNV were similar (**Figure 11B**). Univariate Kaplan–Meier survival curves for CHL1 and PTPRG both indicated that the OS of ccRCC patients was significantly shorter in the low-expression group than in the high-expression group using the Gepia online tool (http://gepia.cancer-pku.cn/index.html) (**Figure 11C**).

### Validation of Correlation Between DEGs and Immune Cell Contents

**Figure 12:**
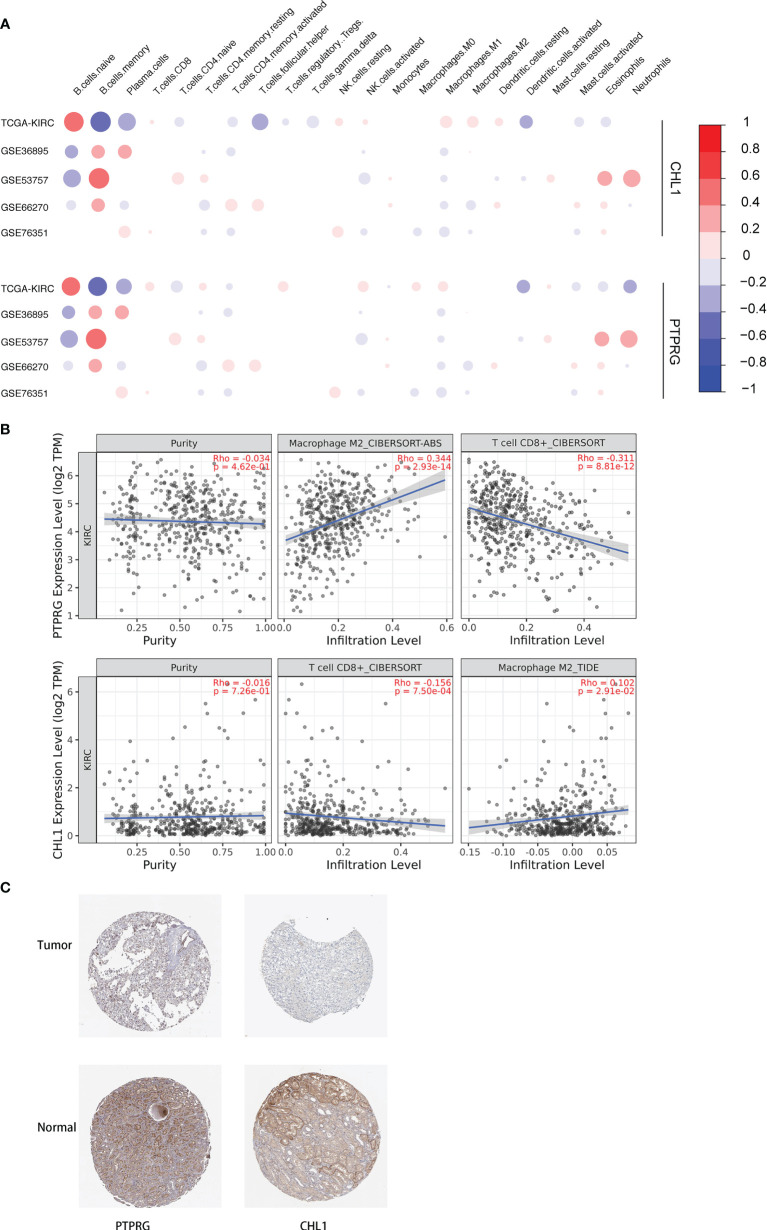
**(A)** Correlation between 22 immune cells relative contents and expression levels of PTPRG and CHL1. The abscissa indicates 22 kinds immune cells while the ordinate represents 5 independent datasets of KIRC. Red means positively related; blue means negatively related; size of circles and shades of colors mean magnitude of correlation. **(B)** Correlation between mRNA levels of PTPRG/CHL1 and relative contents of Macrophage M2 cells and CD8^+^ T cells with online tool TIMER 2.0. **(C)** Immunohistochemical results of PTPRG and CHL1 based on the Human Protein Atlas database.

We conducted a correlation analysis for the expression levels of the PTPRG and CHL1 gene described previously and contents of 22 immune cells, obtaining a correlation pattern similar to that between PD-L1, IFN-γ, and 22 immune cells (**Figure 12A**), which to a certain degree infers the two genes might regulate immune microenvironment *via* pathways associated with PD-L1 and IFN-γ. We also verified the correlation between these two genes and infiltration of CD8^+^ T cells and Macrophage M2 cells in the TCGA-KIRC using the online tool TIMER 2.0 (29) (**Figure 12B**). Using the Human Protein Atlas database, we determined that expression levels of PTPRG and CHL1 were both lower in kidney tumor tissues compared to those in adjacent normal tissues (**Figure 12C**)

### Exploring Intrinsic Roles of CHL1 in ccRCC

**Figure 13:**
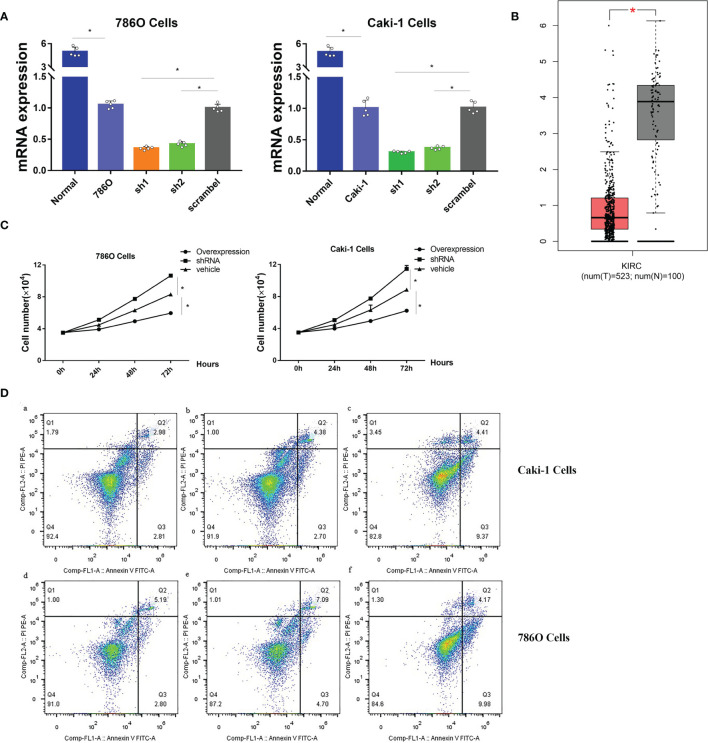
**(A)** mRNA expression profiles of CHL1 in human normal kidney tissues, 786O/sh1/sh2/scramble cells and Caki-1/sh1/sh2/scramble cells. **(B)** mRNA expression profiles of CHL1 between normal tissues and tumor samples in the TCGA-KIRC cohorts with the online tool Gepia. Data represent mean ± s.e.m. Student’s t-test *P <0.05 (two-tailed). **(C)** Proliferation of 786O/Caki-1 cells after CHL1 overexpression and knockdown. The total cell number was counted on days 0, 1, 2, and 3 after transfection. Data represent mean ± s.e.m. (n = 5 per group). One-way ANOVA with the Bonferroni correction for multiple testing, *P <0.05. **(D)** Cell apoptosis was determined by flow cytometry in 786O/Caki-1 cells after transfection for 48 h. **(a, d)**, CHL1 knockdown group; **(b, e)**, vehicle group; **(c, f)**, CHL1 overexpression group.

Since no publications have explored the role of CHL1 in renal cancer *in vitro*, we conducted experiments *in vitro* to investigate the role of CHL1 in tumor growth of 786O cells and Caki-1 cells. The mRNA levels of CHL1 in ccRCC cells were significantly higher than in normal kidney tissues (**Figure 13A**) in line with the results downloaded from the TCGA-KIRC database (**Figure 13B**). Next, we constructed overexpression plasmid and shRNA plasmid (**Figure 13A**) and transfected them into 786O and Caki-1 cells. After transfection, overexpression of CHL1 inhibited tumor cell growth while the knock-down group exhibited accelerated cell viability and a faster cell growth trend compared to the control group (**Figure 13C**). Moreover, flow cytometric analysis indicated that the proportion of apoptotic cells significantly increased following CHL1 overexpression (**Figure 13D**). Altogether, these results highlighted the pivotal role of PTPRG and CHL1 in tumor growth and progression of ccRCC.

### Establishment of Cox-Lasso Regression Analysis Model Based on DEGs Hub

**Figure 14:**
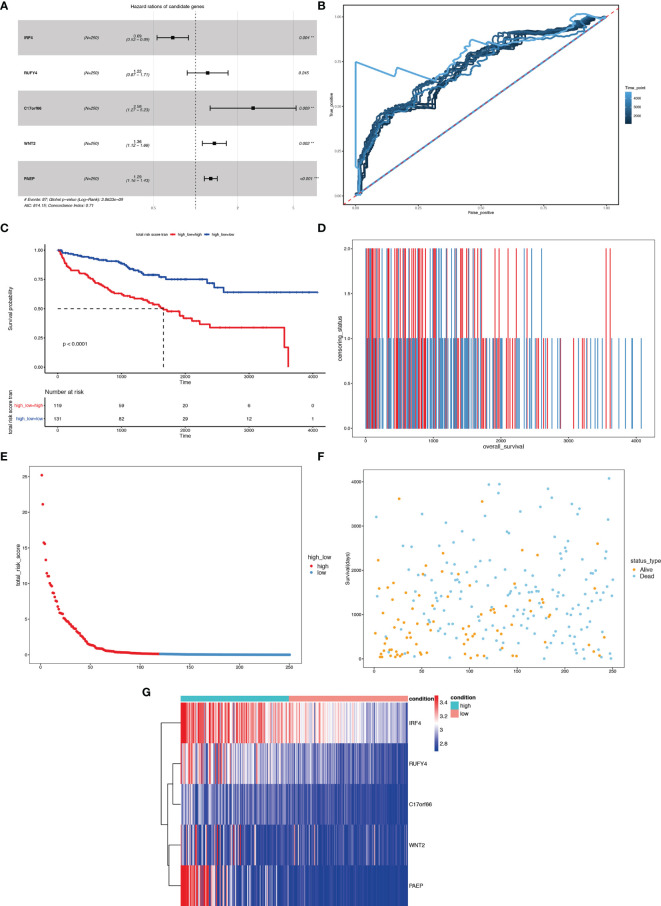
Risk score analysis of the 5-gene signature-based prognostic model in the training group ccRCC patients. **(A)** Five-gene multivariate prognosis model for training group ccRCC patients. **(B)** Time dependent ROC curve analysis exhibits the prognostic performance of the five differentially expressed gene signature-based prognostic model in predicting 1–5-year survival times of the training group ccRCC patients in the TCGA-KIRC cohort. **(C)** Kaplan–Meier curve shows the overall survival of high- and low-risk ccRCC patients in the TCGA-KIRC cohort. **(D**–**F**) Survival bar, risk curve, and survival scatter plot analysis show surviving status of patients of high- and low-risk. **(G)** Heat maps of expression profiles of 5 genes obtained using multivariate Cox regression. **P < 0.01, ***p < 0.001.

**Figure 15:**
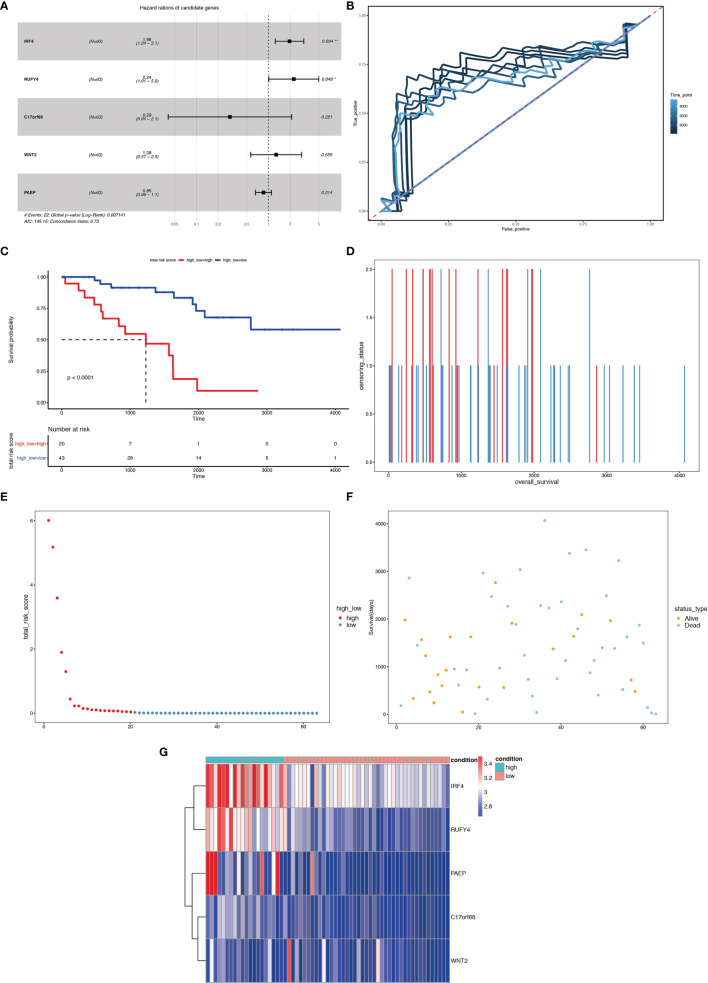
Risk score analysis of the 5-gene signature-based prognostic model in the test group ccRCC patients. **(A)** Five-gene multivariate prognosis model for the test group ccRCC patients. **(B)** Time dependent ROC curve analysis exhibits the prognostic performance of the five differentially expressed gene signature-based prognostic model in predicting 1–5-year survival times of the test group ccRCC patients in the TCGA-KIRC cohort. **(C)** Kaplan–Meier curve shows the overall survival of high- and low-risk ccRCC patients in the TCGA-KIRC cohort. **(D–F)** Survival bar, risk curve, and survival scatter plot analysis show surviving status of patients of high- and low-risk. **(G)** Heat maps of expression profiles of 5 genes obtained using multivariate Cox regression. *P < 0.05, **P < 0.01.

We first carried out a univariate Cox regression analysis to determine the key genes affecting the prognosis of ccRCC patients among the 3,045 differentially expressed genes in A1 and B1 with the following cut-off: | log2(fold change)| >log2(1.5), *p-*value <0.05. Next, further lasso regression analysis and multivariate logistic regression analysis were performed, revealing 22 critical candidates with potential prognostic values. Based on these 22 genes, we established a prognostic model, conducted Kaplan–Meier analysis (**Supplementary Figure 1**) and also applied the risk scores in our previous selected samples (**Supplementary Figure 1**) and detected the expression levels of these 22 genes in samples selected (**Supplementary Figure 1**). However, the prognostic differences between the high and low-risk groups were not significant (*p* = 0.37 >0.05). Therefore, we adopted a more stringent filtration standard and excluded redundant reads (only retain those bigger than 10 and appeared in over 80% of the samples). Analogous to the methods above, we filtered the potential genes based on the DEGs between cluster A1 and B1 but revised the cutoff: | log2(fold change)| >2, *p*-value <0.05. We then divided the 313 patients in the training group into high- and low-risk groups based on the median risk score. After the following univariate Cox regression analysis, lasso regression analysis and multivariate logistic regression analysis, we ultimately determined 5 critical genes (C17orf66, PAEP, WNT2, IRF4, RUFY4) were significantly associated with the prognosis of ccRCC patients in the training group (**Figures 14A, B**). Separately, WNT2, C17orf66, and PAEP were independent risk factors while IRF4 was independent protective factors (*p* 0.01). Kaplan–Meier survival curve analysis indicates that the OS was significantly shorter for the high-risk group ccRCC patients compared to the low-risk group ccRCC patients (**Figure 14C**). The risk score of each patient was calculated with the following formula: Risk score = (−0.0398 ∗ Exp IRF4) + (0.2617 ∗ Exp WNT2) + (0.0556 ∗ Exp RUFY4) + (0.5507 ∗ Exp C17orf66) + (0.1441 ∗ Exp PAEP). The ROC curve of the risk score model indicates moderate to good performance in predicting one-year OS (AUC = 0.715), three-year OS (AUC = 0.728), and five-year OS (AUC = 0.814) (**Figure 14D**). **Figures 14E** **–G** show the surviving status of patients in cluster A1, B1, the risk curve analyses and the expression profiles of the five genes in the high- and low- risk group ccRCC patients. We then calculated the risk scores of the test group patients using the same prognostic risk score formula and assessed the predictive performance of the prognostic risk model. The OS was significantly shorter for the ccRCC patients in the high-risk group compared to the low-risk group ccRCC patients (**Figure 15**).

## Discussion

Further investigations about the targeted ccRCC population potentially benefited from ICI therapy and more in-depth elucidation of the underlying mechanisms governing the profit differentiation are desperately needed based on the strikingly increasing application of ICI therapy among ccRCC patients. This study initially stratifies tumors into several subgroups with different immune cell infiltration in 5 ccRCC cohorts. An analysis of the mutational landscape and expression differentiation helps construct DEG hub, reveals genetic differences among immune subgroups with higher or lower immune infiltration and identifies PTPRG and CHL1 as vital key nodes of the expression regulating network. Finally, we established a prognostic model predicting the targeted kidney cancer patients suitable for ICI therapies.

Based on the relatively satisfying oncological outcomes in the recent high-quality trials (30–32), the European Association of Urology (EAU) provided a strong rating and category 1 recommendation for ICI-based therapy in metastatic RCC (33). According to the latest research by Wu et al. (34), the adverse events of immune checkpoint inhibitor therapy for kidney cancer were significantly less reported than those in urothelial cancer or prostate cancer, proposing the full-fledged rather than experimental roles of ICB therapies in RCC. Beuselinck et al. (35) defined four subsets of ccRCC patients (ccRCC1–ccRCC4) according to their clinical responses to the TKI sunitinib and named ccRCC4 as a no-response subtype with high expression of PD-1 and suppressive immune microenvironment while ccRCC2 as an improved-response subtype with upregulation of angiogenesis-related genes. For comparison, most cases in subtype A1 in our study can be classified as ccRCC4 while the majority of cases in subtype B1 can be classified as ccRCC2.

Our study mainly differs from recently published ccRCC studies in that we performed an integrated multi-omics analysis to underline the mutational landscape and epigenetic patterns responsible for the immune classifications in ccRCC. On top of that, we established a regression model to predict the prognosis of ccRCC patients based on five immune-linked candidate genes.

Significant copy numbers deletion variation of chromosome 3p was a prominent feature between clusters A1 and B1 in ccRCC. Tsuyukubo et al. found that 3p24.3 mixed type was inversely correlated with the presence of metachronous metastasis and can predict a favorable prognosis in ccRCC (36). Analogous to our study, the distribution difference in chromosome 3p instead of individual gene alterations might explain the variability of the immune cell infiltration and even immunophenotypes among the ccRCC clusters.

Specifically in our study, we also discovered the differential distribution in chromosome 5p including some DEGs believed to regulate immune escaping and immune co-inhibition like CD74 (37, 38) and EGR1 (39) might potentially serve as key nodes in constructing the differentially shaped immune landscape of ccRCC. Moreover, our results revealed mir-215, mir-155, SOCS1, CD274, and SOX11 could play intriguing roles in the classification of ccRCC. MiR-215 has been shown to play an important role in the occurrence, development, and prognosis of many malignant tumors by regulating cell proliferation, metastasis, apoptosis and drug resistance (29–31). SOCS1, CD274, and SOX11 associated with miR-155 and miR-215 in the regulation hub suggested exerting vital functions in tumor immunology, whether through the miR-155, miR-215 pathway or not (32–35).

On top of genetic alterations, epigenetic modifications also played a vital role in forming the ‘targeting immunity framework’ to regulate the delicate balance of immune homeostasis, priming, training, tolerance and even contribute to immune evasion in cancer (40), especially when manifesting as methylation (41). Klümper and colleagues reported DNA methylation of lymphocyte activating 3 strongly correlated with signatures of distinct immune cell infiltrates and survival in KIRC (42).

In this study, we conducted multi-omics research integrating transcriptomic, genetic, and epigenetic analyses to unravel the key components determining the immunophenotypes and corresponding response (positive or negative) to ICB therapy. We subsequently identified some significant hub genes through the construction of the DEGs–miRNA–lncRNA–mRNA regulation hub centered at PTPRG and CHL1.

Protein tyrosine phosphatase receptor gamma (PTPRG), a member of the protein tyrosine phosphatase (PTP) superfamily of enzymes capable of removing phosphate groups from phosphorylated tyrosine residues bringing an equilibrium status in normal populations, were described as a tumor suppressor in various neoplasms. Previous studies have revealed its crucial role in tumors of the lung (43), ovarian (44), nasopharyngeal carcinoma (45), and chronic myelogenous leukemia (46). Likewise, PTPRG was also identified as a suppressor gene in RCC (47). Arimura (48) reported PTPRG was least expressed in immune cells, B cells, in particular, suggesting it might be engaged in suppressing tumor growth and metastasis through regulation downstream signals and actions of immune cells. The close homolog of L1 (CHL1, also referred to as CALL), is known as a cell adhesion molecule of the immunoglobulin (Ig) superfamily (49). CHL1 has also been reported associated with the suppression of neoplastic growth and metastasis in various tumors (50–52). Our results supported its suppressing role in the proliferation and also promoting apoptosis in ccRCC. Combing previous researches with our results, we propose that low expression and high methylation of PTPRG and CHL1 facilitate the formation of high cytotoxic immune phenotypes thus leading to better response to ICB therapy.

Finally, we constructed a Cox-Lasso logistic regression prognosis model based on differentially expressed genes between clusters A1 and B1 of the KIRC cohort. Five candidate genes were screened out and visualized in Kaplan–Meier survival curves, including widely recognized factors of immune regulation (53, 54). Our study included the following limitations. First, the data we utilized for analysis were all downloaded from databases like the GEO and TCGA rather than obtained from our original samples. Second, our study gained results mainly based on the difference of the two most prominent immune sub-clusters but we cannot ignore the effects of some immune subtypes even in low abundance. Finally, we did not acquire meaningful results when investigating the cohorts of the ccRCC patients who have received ICB therapy due to the limited samples available for the public (**Supplementary Figure 2**).

In conclusion, our study divided ccRCC into distinct immune clusters and thereby identified two DEGs, namely, PTPRG and CHL1, which were validated to play a crucial role in inhibiting tumor growth and might be responsible for the formation of distinct immune clusters in ccRCC. We also constructed a prognosis model based on the DEGs between high immune-infiltrating cluster and low immune-infiltrating cluster and we hope ccRCC patients before or during ICI therapy could profit from this immunophenotypes-associated risk stratification model.

## Data Availability Statement

The datasets presented in this study can be found in online repositories. The names of the repository/repositories and accession number(s) can be found in the article/**Supplementary Material**.

## Author Contributions

DP, ZH and XZ contributed to the conception and design of the study. XZ and DP performed the collection, investigation, curation and formal analysis of data. LL wrote the first draft of the manuscript. DP, ZH, and XZ reviewed the manuscript and wrote sections of the manuscript. All authors listed have made a substantial, direct, and intellectual contribution to the work and approved it for publication.

## Conflict of Interest

The authors declare that the research was conducted in the absence of any commercial or financial relationships that could be construed as a potential conflict of interest.

## Publisher’s Note

All claims expressed in this article are solely those of the authors and do not necessarily represent those of their affiliated organizations, or those of the publisher, the editors and the reviewers. Any product that may be evaluated in this article, or claim that may be made by its manufacturer, is not guaranteed or endorsed by the publisher.

## Supplementary Material

Supplementary Figure 1 Prognostic model based on DEGs between A1 and B1 with the cut-off set as | log2(fold change)| > log2(1.5), *p-*value < 0.05.

Supplementary Figure 2 Prognostic model based on intersection of DEGs between A1 and B1 and significant genes associated with reponse to PD1 therapies in cohort GSE67501(n=11).

Supplementary Table 1 Genes with significant CNV variations between A1 and B1.
