# Supplementary figures and images for: Integrated Multi-Omics Analysis Identified PTPRG and CHL1 as Key Regulators of Immunophenotypes in Clear Cell Renal Cell Carcinoma(ccRCC)
Source: Front Oncol. 2022 Mar 30;12:832027. doi: 10.3389/fonc.2022.832027 (PMC9005830; doi:10.3389/fonc.2022.832027)

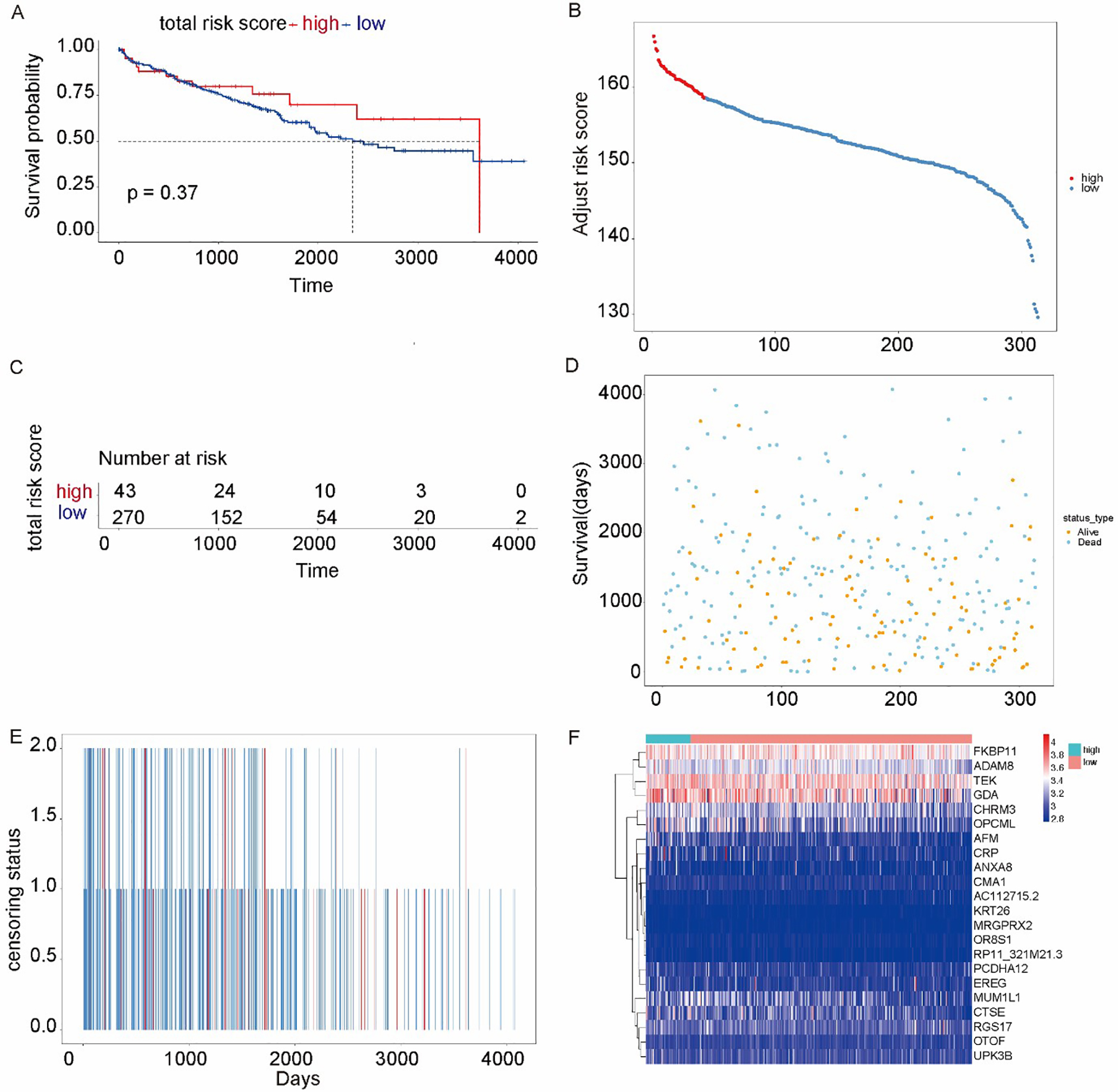

Supplement: Supplementary Figure 1 — Prognostic model based on DEGs between A1 and B1 with the cut-off set as | log2(fold change)| > log2(1.5), p-value < 0.05. [file Image_1.jpeg]

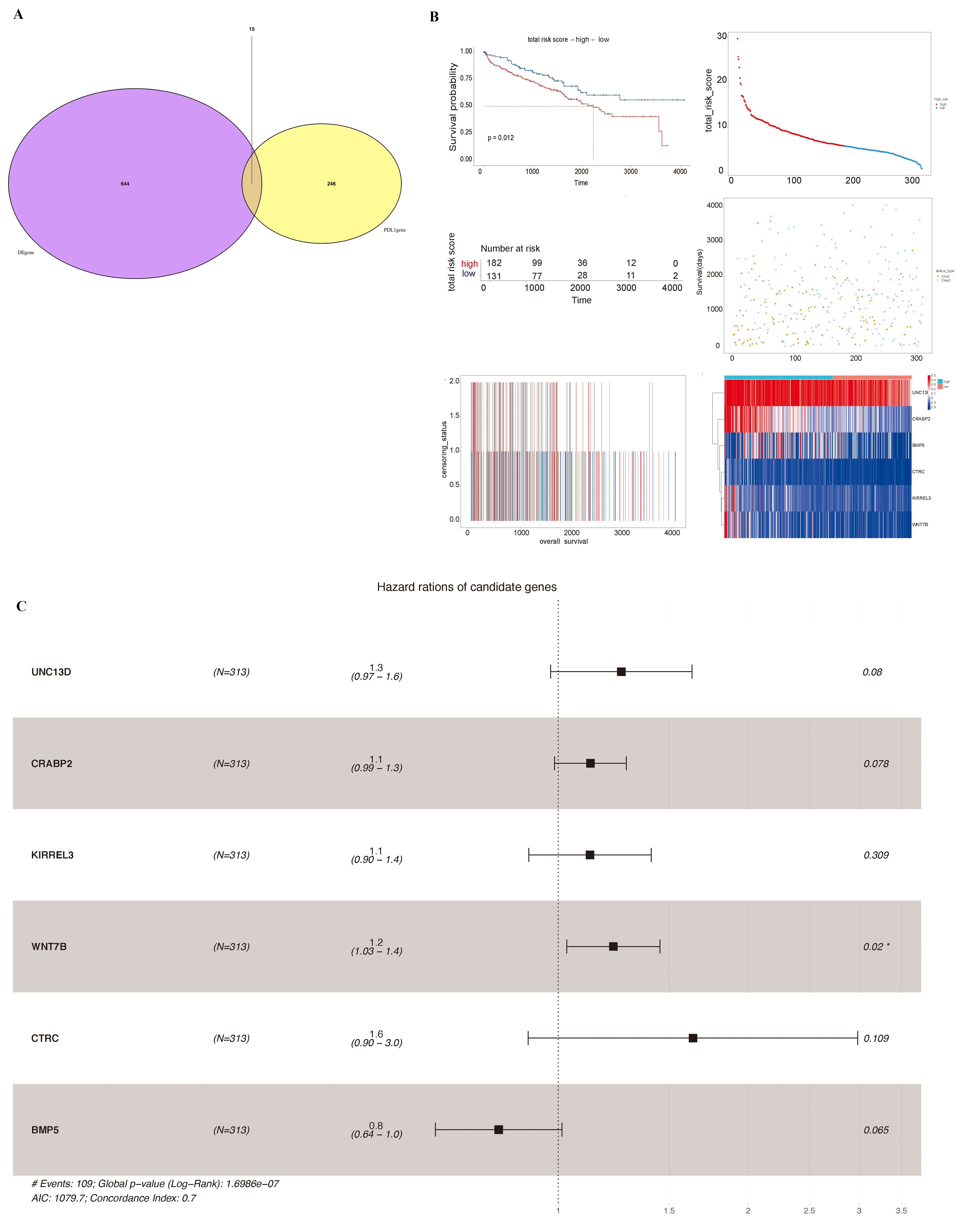

Supplement: Supplementary Figure 2 — Prognostic model based on intersection of DEGs between A1 and B1 and significant genes associated with reponse to PD1 therapies in cohort GSE67501(n=11). [file Image_2.jpeg]
