# Supplementary material for: Integrated Multi-Omics Analysis Identified PTPRG and CHL1 as Key Regulators of Immunophenotypes in Clear Cell Renal Cell Carcinoma(ccRCC)
Source: Front Oncol. 2022 Mar 30;12:832027. doi: 10.3389/fonc.2022.832027 (PMC9005830; doi:10.3389/fonc.2022.832027)
Supplement: Supplementary file 4 [file DataSheet_1.docx]

**Construction of overexpression/shRNA plasmid**

Full-length CHL1 was polymerase chain reaction (PCR)-amplified using the following primers (F: 5’-CTA GCT AGT TAA TTA AGG ATC CGC CAC CAT GGA GCC GCT TTT ACT TG-3’; R:5’-CCC TCG AGG CCT GCA GGA ATTC TTA TGC CCG AAG GGG AAA AG-3’). CHL1 cDNA was inserted into the expression vector, pMYs-IRES-Puro (pMYs-IP) (Cell Biolabs, San Diego, CA, USA) using infusion cloning enzymes (Vazyme, One-step cloning kit). Plasmids with correct sequences were digested with restriction enzymes (Bamh1 and Ecor1). For short hairpin RNA (shRNA)-mediated Dhx15 knockdown experiments, the primer for sh1 was: F: 5’-CCG GCA GCA ATA TTA GCG AGT ATA TCT CGA GAT ATA CTC GCT AAT ATT GCT GTT TTT G-3’, R: 5’-AAT TCA AAA ACA GCA ATA TTA GCG AGT ATA TCT CGA GAT ATA CTC GCT AAT ATT GCT G-3’; the primer for sh2 was: F: 5’-CCG GCG TCC ATT GAT ACA AAC CAA ACT CGA GTT TGG TTT GTA TCA ATG GAC GTT TTT G-3’, R: 5’-AAT TCA AAA ACG TCC ATT GAT ACA A AC CAA ACT CGA GTT TGG TTT GTA TCA ATG GAC G-3’; the primer for scramble was : F: 5’-GAT CCA ACA AGA TGA AGA GCA CCA ACT CGA GTT GGT GCT CTT CAT CTT GTT GTT TTT G-3’, R: 5’-AAT TCA AAA ACA ACA AGA TGA AGA GCA CCA ACT CGA GTT GGT GCT CTT CAT CTT GTT G-3’. shRNA and scramble nucleotides were inserted into the pMXs-IRES-Puro (pMXs-IP) vector (Cell Biolabs, San Diego, CA, USA) using infusion cloning enzymes (Vazyme, One-step cloning kit).

**Real-time PCR analysis**

To analyze the expression of CHL1 in 786O cells, Caki-1 cells and HK-2 cells (representing normal kidney cells), we performed qPCR using the following TaqMan assays (Invitrogen, Carlsbad, CA, USA). Primer: F: 5’-AGT CCA AGT TGC CTT TCC CTT-3’, R: 5’-AGT CCA CGA AAA TGT TGG TTC T-3’; Gapdh primer: F: 5’-TGA CCA CAG TCC ATG CCA TC-3’; R: 5’-CAT ACC AGG AAA TGA GCT TGA-3’. The relative expression ratio was calculated by the 2^−ΔΔCT^ method.
